# Temperature dependence of afterglow in zirconia and its optically-stimulated luminescence by bone-through irradiation for biological temperature probe

**DOI:** 10.1038/s41598-020-58979-4

**Published:** 2020-02-10

**Authors:** Masaharu Ohashi, Yoshihiro Takahashi, Nobuaki Terakado, Noriko Onoue, Tsuyoshi Shinozaki, Takumi Fujiwara

**Affiliations:** 1 Department of Applied Physics, Graduate School of Engineering, Tohoku University, Aoba 05, Aoba-ku, Sendai 980-8579 Japan; 2 Department of Cardiovascular Medicine, National Hospital Organization, Sendai Medical Center, 8-8, 2-chome, Miyagino, Miyagino-ku, Sendai, Miyagi 983-8520 Japan

**Keywords:** Sensors and biosensors, Optical sensors

## Abstract

Development of minimally invasive and site-selective biological temperature sensing is quite important in medical field. This study presents a novel temperature sensing technique based on afterglow and optically-stimulated luminescence (OSL). The dependence of afterglow photoluminescent intensity on the environmental temperature of zirconia (ZrO_2_) phosphor is examined to validate its use as a sensing probe. In addition, assuming the measurement in deep-part of human body, we have applied the information gathered from our validation to observe OSL from the ZrO_2_ by irradiation with near-infrared laser through a bone sample. This study demonstrates an alternative medical application of phosphor, and introduces an elemental-technology for the temperature sensing.

## Introduction

Body temperature is main vital sign, which is closely related to immune system and metabolism. A technique for biological temperature monitoring is a powerful tool for medical treatment and monitoring human health. In addition, targeted temperature management has the potential to reduce the risk of brain dysfunction after a head injury and cardiopulmonary arrest. Although clinical thermometers and thermography are non-invasive, the methods measure the temperature on the surface of body. The gold standard for measuring temperature in critically ill patients is the Swan-Ganz catheter (Suppl. Fig. S1). The catheter must be inserted into the body in order to monitor the temperature at the desired location. Consequently, there is a need for accessible and minimally invasive temperature sensing methods which can monitor local internal temperature.

Optical/spectroscopic measurement is recognized to be a minimally invasive and contactless method, and particularly, fluorescent and afterglow phenomena have been attempted to utilize for sensing-/imaging-applications so far^1–5^. Quantum-dots and fluorescent polymers have been proposed as temperature sensors for cell-imaging due to the relationship between temperature and fluorescence intensity/lifetime^1,2^. The observation of these effects is only possible with the magnification of a microscope. Chermont *et al*. developed a bio-imaging technique using afterglow phosphor of Eu^2+^, Dy^3+^, Mn^2+^-tri-doped Ca_0.2_Zn_0.9_Mg_0.9_Si_2_O_6_^6^. Their study results inspired us to use the afterglow phenomenon to measure the temperature within the human body. In this manuscript, the fundamental measurements necessary to validate the use of the decay curve in the afterglow phosphor to measure temperature are presented. In addition, optically-stimulated luminescence (OSL) phenomena was also considered as irradiation of near-infrared (NIR) laser light would enable site-selective photoluminescence (PL), making it possible to obtain temperature information at internal sites in the human body.

## Conception and Fundamental Principle of Temperature Measurement

**Figure 1:**
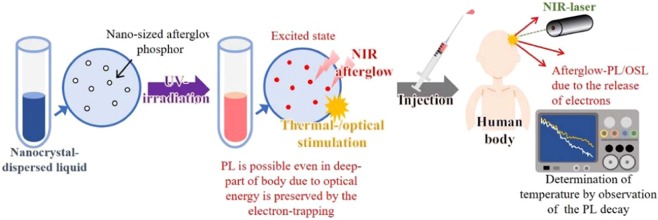
Temperature sensing concept for measuring local internal temperature using afterglow phosphor.

We propose a new concept for minimally invasive biological temperature sensing method using afterglow phosphor^7^. In Fig. 1, we display a rough sketch of our concept: Afterglow originates in the recombination between holes and electrons that are trapped at a metastable site and subsequently released thermally. Since the release of electrons depends on thermal excitation, the environmental temperature determines the afterglow lifetime (or slope of the afterglow curve). If only one kind of the electron-trapping site is presented in afterglow phosphor, its temperature dependence on the intensity of afterglow PL follows to simple exponential relationship^8^:

1 $$I(t)/I(0)\propto \exp (-at),$$

2 $$a=s\cdot \exp (-\frac{E}{{k}_{{\rm{B}}}T}),$$

where *I*(*t*) is the afterglow PL intensity, *I*(0) is the initial intensity, *t* is the time, *a* is the probability of thermal activation of a trapped electron to the conduction band, *E* is the thermal activation energy of an electron in the trap, *s* is the frequency factor, *k*_B_ is the Boltzmann constant, and *T* is the temperature. Since the *a* corresponds to an inverse of lifetime for the PL, observing the temperature dependence on the *a* (=1/τ), we obtain the activation energy. As a result, we are able to estimate the environmental temperature of the phosphor from the afterglow measurement. In order to estimate the temperature, a suspension consisting of afterglow nanophosphor is prepared and is excited prior to injection into the region of interest. After injection, afterglow PL is detected from the outside of body to obtain information about the internal temperature. For the afterglow, the PL wavelength should be in the NIR region, which is called to “biological window,” (~650–1100 nm)^9^. Alternatively, the trapped-electrons can be released optically, for instance using irradiation of a NIR-laser, and then the recombination provides the luminescence, i.e., OSL. Therefore, we believe that OSL can also be used to obtain temperature information. Since a laser is a coherent light-source, site-selective temperature sensing at any location in the body should be possible using external irradiation with a NIR-laser.

### Temperature measurement based on afterglow zirconia phosphor

In this study, we have focused on afterglow zirconia (ZrO_2_) as a sensing probe. ZrO_2_ is an accessible material and possesses a high chemical-stability and bio-compatibility^10^. In addition, the size, morphology, physical and optical properties can be controlled by choice of appropriate synthetic conditions and dopants^11,12^. Pure ZrO_2_ has different polymorphs, and the monoclinic phase exhibits a remarkable PL^13^. Furthermore, monoclinic ZrO_2_ acquires a prominent afterglow property through the introduction of oxygen-defects, which are activated by the annealing^14,15^. Iwasaki *et al*. have reported that thermally-treated ZrO_2_ powder shows pronounced afterglow property^15^. Therefore, we selected the thermally-treated ZrO_2s_ powder as our sensing probe (see Methods).

**Figure 2:**
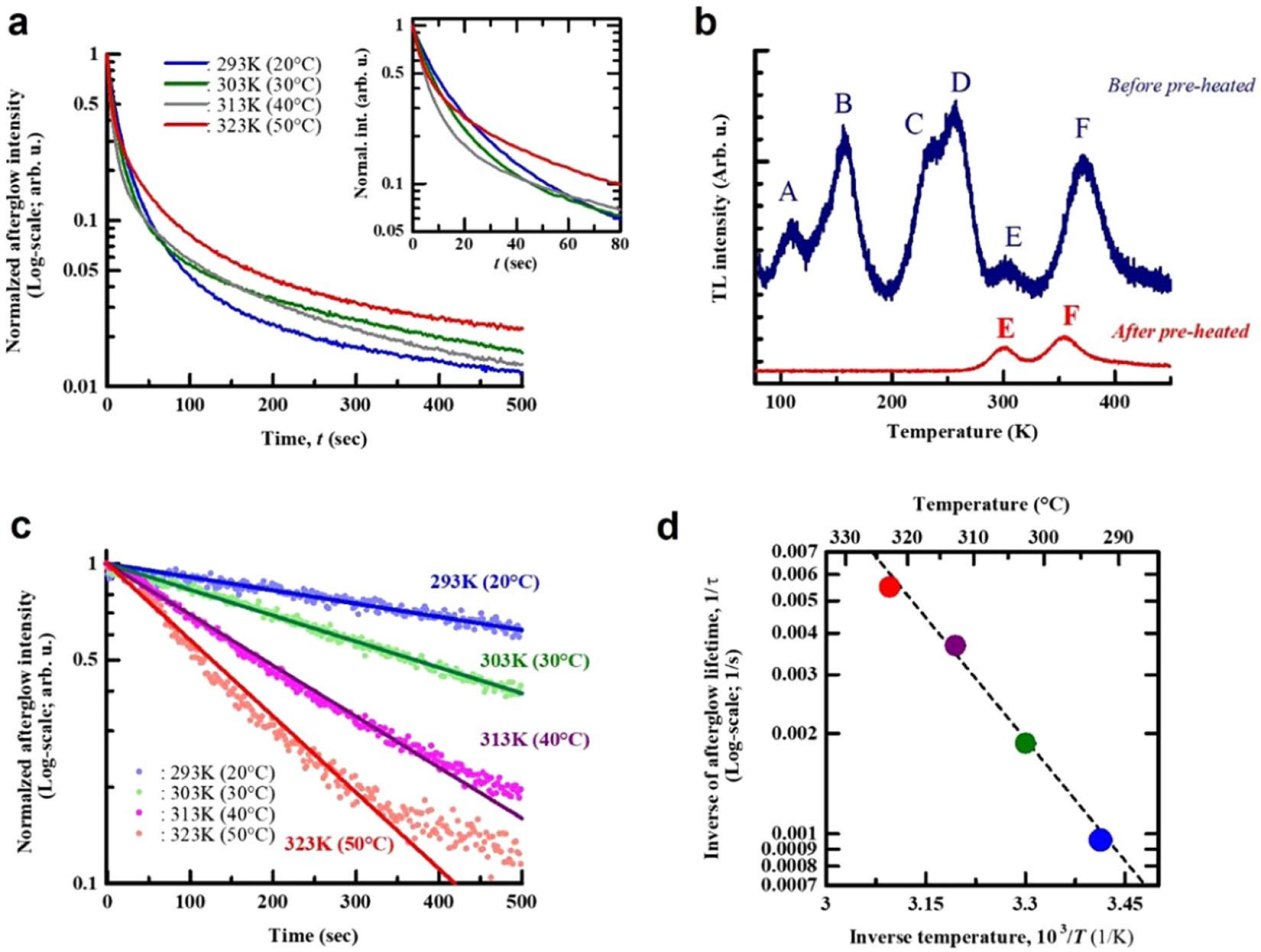
(**a**) Afterglow decay curves of ZrO_2_ sample at different environmental temperatures, and (**b**) TL spectra in the samples before and after pre-heat-treatment (de-trapped) at 273 K for 2.5 h. (**c**) The decay curves of the de-trapped ZrO_2_ sample at different temperatures and (**d**) the obtained values of probability as a function of inverse temperature. The dashed line corresponds to the fitting result, and its coefficient of determination is estimated to be *R*^2^ = 0.977.

In Fig. 2, we show the afterglow PL and thermoluminescent (TL) properties of the ZrO_2_ sample. Excitation and detected-afterglow wavelength were λ_exc_ = 280 nm and λ_emi_ = 480 nm, respectively. When the decay curve is plotted on a semi-log-y graph, a linear relationship between time and intensity should be obtained. The slope should be steeper for higher environmental temperatures, show faster decay, according to the Eqs. (1)–(2). However, the linearity was not observed at any temperature [Fig. 2(a)]. The decay curves showed the complex behavior at times less than 200 s when reversal of the intensity and intersection of the decay curves is seen. The TL spectrum revealed six distinguishable peaks (indicated by A to F) in the temperature range tested [Fig. 2(b)]. The decay curve and TL spectrum indicates the presence of various kind of electron-trapping sites in the ZrO_2_ sample, suggesting that the plural trapping sites cause the non-linearity and complicated decay curves. In order to release the electrons form the trapping sites corresponding to the TL peaks A–D appearing below room temperature (RT), so-called de-trapping, a pre-heating process at 273 K for 2.5 h before UV-excitation for 1 h was conducted prior to TL measurement. As the result, the peaks A–D vanished and indicating the release of electrons at the traps which are active below RT. The pre-heating process successfully produced a de-trapped ZrO_2_ sample [red curve, Fig. 2(b)]. The de-trapped sample exhibited the expected response (Eqs. (1)–(2)) of linear decay with slopes that increased with increasing temperature [Fig. 2(c)]. The response at 323 K (50 °C) deviated from the theoretical line. This departure was probably due to a partial electron-release from a deeper-trapping site corresponding to the peak F with peak at ~353 K. The pre-heating process is an effective technique to minimize the non-linearity in the decay curve. Using the Arrheniusian approach [Fig. 2(d)] the activation energy for afterglow PL related to the peak E for the pre-heated samples was found to be *E*~0.48 eV. Thus, we demonstrated the fundamental temperature sensing principle based on the afterglow measurement in the ZrO_2_ sample.

### Optically-stimulated luminescence by bone-through light

Since NIR can minimize its absorption loss for the tissue texture (i.e., biological window), laser irradiation with a NIR wavelength enables us to stimulate the PL and to obtain temperature information based on the OSL. Until now, OSL phosphor has been extensively studied for the application in detection of charge particle, and particularly the ZrO_2_ is a candidate for the OSL phosphor toward scintillator/dosimeter material because of being chemically and thermally stable^16–19^. Assuming the observation of a body part covered by bone (e.g., brain), it is necessary to stimulate the luminescence via bone by external laser irradiation. This is a significant issue that should be clarified for the OSL application in human body. In order to confirm that stimulation via bone was possible, we prepared a simulated bone sample and examined the OSL from the ZrO_2_ sample.

**Figure 3:**
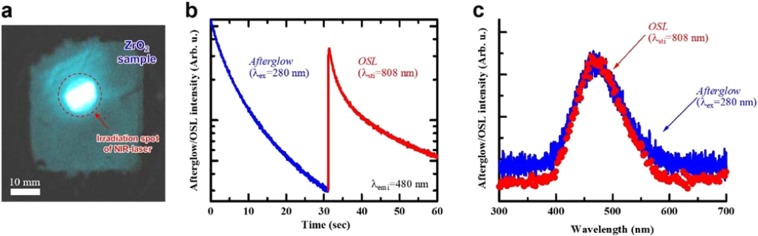
(**a**) Visual demonstration of the OSL in ZrO_2_ sample, which was excited by UV-irradiation in advance. The bright region (encircled by dashed line) corresponds to the irradiation spot. (**b**) Intensity of the afterglow PL and subsequent OSL as a function of time. (**c**) The spectra of afterglow PL and OSL (stimulated by NIR-laser with λ_sti_ = 808 nm).

In Fig. 3, we show the OSL properties of the ZrO_2_ sample. By irradiation of the NIR-laser light (λ_sti_ = 980 nm), we visually confirmed the bluish PL spot in the irradiated area [Fig. 3(a)]. The excited ZrO_2_ sample exhibited a decrease in PL with time (i.e., afterglow), and a sudden increase in the PL intensity and its subsequent decay were confirmed as the ZrO_2_ sample was irradiated with NIR-laser (808 nm) [Fig. 3(b)]. Both the OSL and PL spectrum shown in [Fig. 3(c)] have a broad band with peak at ~480 nm, which is similar to the usual PL spectrum (see Suppl. Fig. S2) demonstrating obvious OSL in the ZrO_2_ sample.

**Figure 4:**
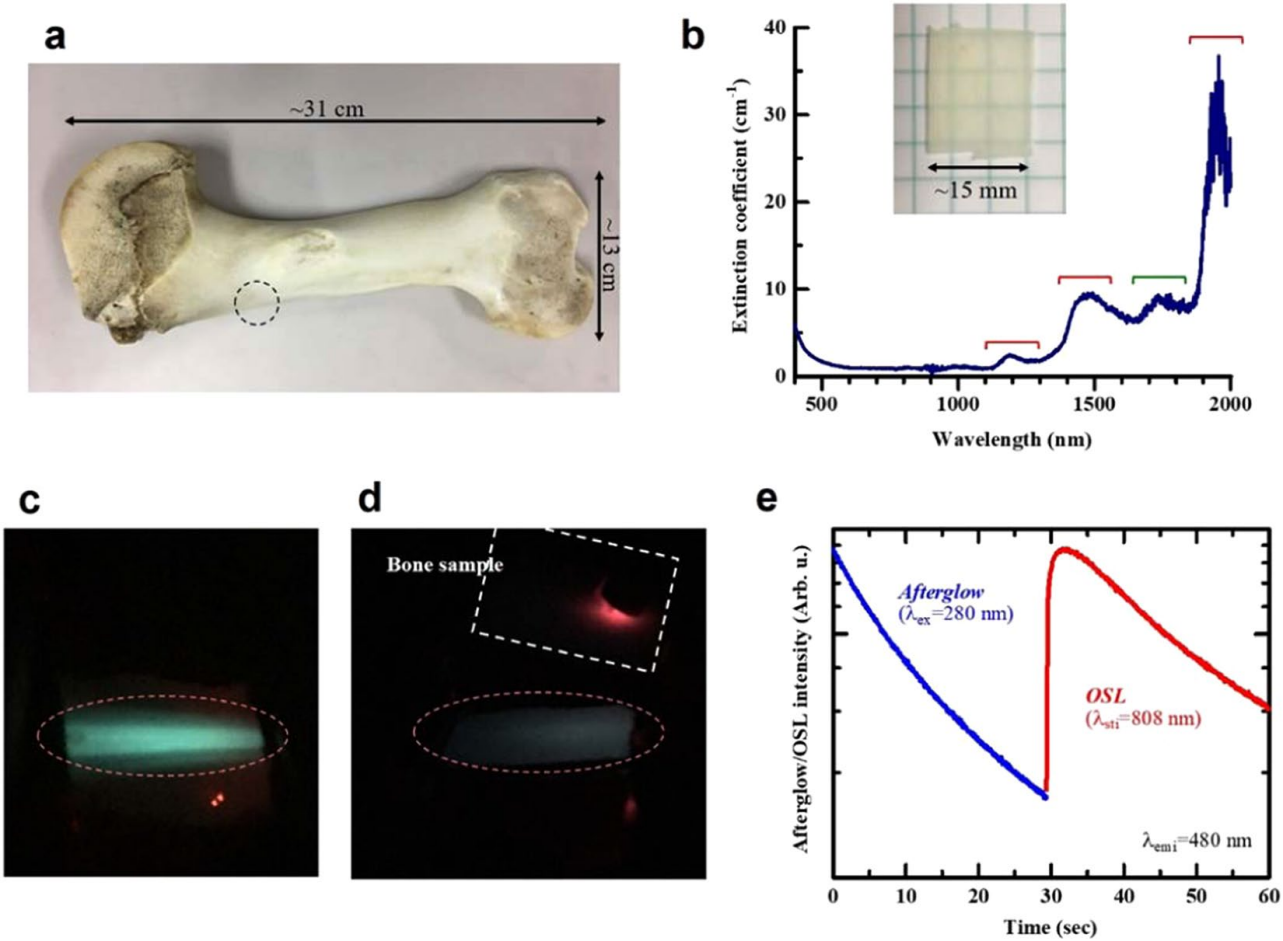
(**a**) Cattle femur bone used in this study. The encircled region corresponds to the position, at which the bone sample was obtained. (**b**) optical extinction spectrum of the simulated bone sample (inset). The bands indicated by red and green bars are attributed to absorption due to water and lipid, respectively, after Bashkatov *et al*.^20^. (**c**) OSL from the ZrO_2_ sample observed by the irradiation of NIR-laser light without the bone sample. (**d)** The OSL from the ZrO_2_ sample stimulated by the laser light through the bone sample. Dashed line indicates the contour of bone sample, which was settled in front of the NIR-laser diode. The blue emission of OSL could be observed in the ZrO_2_ sample region as well as the observation without the bone sample as indicated by dashed red circles. (**e**) The intensity of the afterglow PL and subsequent OSL as a function of time in the ZrO_2_ sample stimulated by the laser light through the bone sample.

In Fig. 4, we show the results of OSL by irradiation of NIR through the simulated bone sample. We have cut and polished a rectangular section (dimension: ~20 × 15 × 0.5 mm^3^) of bone from a cattle femur [Fig. 4(a,b)]. The spectral features of the optical extinction spectrum for the cattle bone was quite similar what has been reported for human bone^20^: the broad absorption bands are attributed to water and lipid molecules [Fig. 4(b)]. An optically-transmittable region in ~650–1000 nm was found, leading us to speculate that OSL by NIR light through bone is possible. The Raman spectra in the range of 300–3000 cm^−1^ (Suppl. Fig. S3) also resembled the spectral patterns reported for human bone^21,22^. The Raman bands could be assigned to vibration modes related to phosphate, amide, and C–H bonds. These similarities show that the cattle femur bone is an appropriate animal model for human bone.

Observation of the OSL by NIR-laser irradiation through the simulated bone sample was performed and we could visually confirm bluish PL from the ZrO_2_ sample [Fig. 4(c,d)]. In addition, the sudden increase and subsequent decay of the PL intensity was also confirmed [Fig. 4(e)], OSL by the bone-through irradiation. The intensity of OSL presumably exhibits the similar temperature dependence to that of afterglow phenomenon because the OSL also originates from the release of electrons in trapping sites. Therefore, estimation of the environmental temperature is could be possible. The combination of OSL and afterglow phosphor by means of external stimulation with NIR-laser is a promising technique for site-selective temperature measurement. Indeed, the investigation of temperature dependence of OSL in the ZrO_2_ sample is now in progress.

## Discussion

We have proposed a new concept for internal temperature measurement using a harmless and accessible inorganic crystal, afterglow ZrO_2_, as a sensing probe. In this study, the fundamental principle of temperature sensing based on afterglow/OSL and its feasibility were demonstrated. Again, we want to stress that the OSL by means of NIR-laser enables us to measure the localized temperature at internal locations (e.g., the brain) non-invasively and site-selectively. The temperature dependence of OSL intensity and the red-shift of afterglow PL and OSL in ZrO_2_ phosphor must be investigated. The authors’ group has already succeeded in fabricating ZrO_2_ phosphor showing afterglow PL in red region^23^. While this fundamental study leaves some important issues unresolved, this work is a novel and promising application of phosphor in the medical field.

## Methods

Commercial reagent-grade ZrO_2_ powder (purity: 99.9%, Soekawa Chem. Co., Ltd., white powder with size of ~3 um) was thermally-treated at 1400 °C. The heating rate was 10 K/min and the target temperature was maintained for 6 h in an electric furnace under atmospheric conditions. After the treatment, the ZrO_2_ powder was cooled in the furnace, producing defect-activated ZrO_2_^15^. The ZrO_2_ sample could be identified to be monoclinic by means of a powder X-ray diffraction (XRD; Cu-Kα) analysis. The ZrO_2_ sample exhibited the broad PL and PLE bands with peaks at ~480 nm and ~280 nm, respectively (Suppl. Fig. S2). In addition, the visible PL and bluish afterglow was also confirmed. The PL and afterglow features were identical to the features previously reported^15^ indicating that the ZrO_2_ prepared in the earlier study was properly reproduced. A cattle femur bone that is commercially available is used as the simulated human bone sample in this study. Length and width of the cattle bone are approximately 31 cm and 13 cm, respectively [Fig. 4(a)].

The PL and PL excitation (PLE) spectra, and afterglow PL intensity as a function of time (decay curve) was obtained using a spectrofluorometer with a xenon lamp as the excitation source. The environmental temperature of the ZrO_2_ sample during decay curve measurement was controlled by a cryostat (Temperature range: 293–323 K; 20–50 °C). Afterglow PL intensity as a function of temperature (TL spectrum) was also measured by means of the spectrofluorometer conjugated with the cryostat (Temperature range: 77–450 K, heating rate: 1 K/min). Prior to the TL measurement, the ZrO_2_ sample was subjected to UV-excitation (280 nm) for 1 h. The cattle-bone sample’s optical properties were characterized using a spectrophotometer with an integrating sphere and Raman scattering spectroscopy (Excitation wavelength: 532 nm).

## Supplementary information

Supplementary information
