# Supplementary material for: Temperature dependence of afterglow in zirconia and its optically-stimulated luminescence by bone-through irradiation for biological temperature probe
Source: Sci Rep. 2020 Feb 10;10:2242. doi: 10.1038/s41598-020-58979-4 (PMC7010703; doi:10.1038/s41598-020-58979-4)
Supplement: Supplementary file 1 — Supplementary information [file 41598_2020_58979_MOESM1_ESM.docx]

**Temperature dependence of afterglow in zirconia and its optically-stimulated luminescence by bone-through irradiation for biological temperature probe**

Masaharu Ohashi^1^, Yoshihiro Takahashi^1,†^, Nobuaki Terakado^1^, Noriko Onoue^2^, Tsuyoshi Shinozaki^2^ and Takumi Fujiwara^1, ‡^

^1^*Department of Applied Physics, Graduate School of Engineering, Tohoku University, Aoba 05, Aoba-ku, Sendai, 980-8579, Japan*

^2^*Department of Cardiovascular Medicine, National Hospital Organization, Sendai Medical Center, 8-8, 2-chome, Miyagino, Miyagino-ku, Sendai, Miyagi 983-8520, Japan*

**Supplementary Information**

**
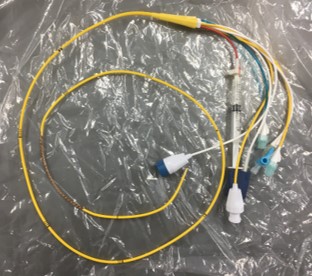
**

**Fig. S1.** Swan-Ganz catheter.


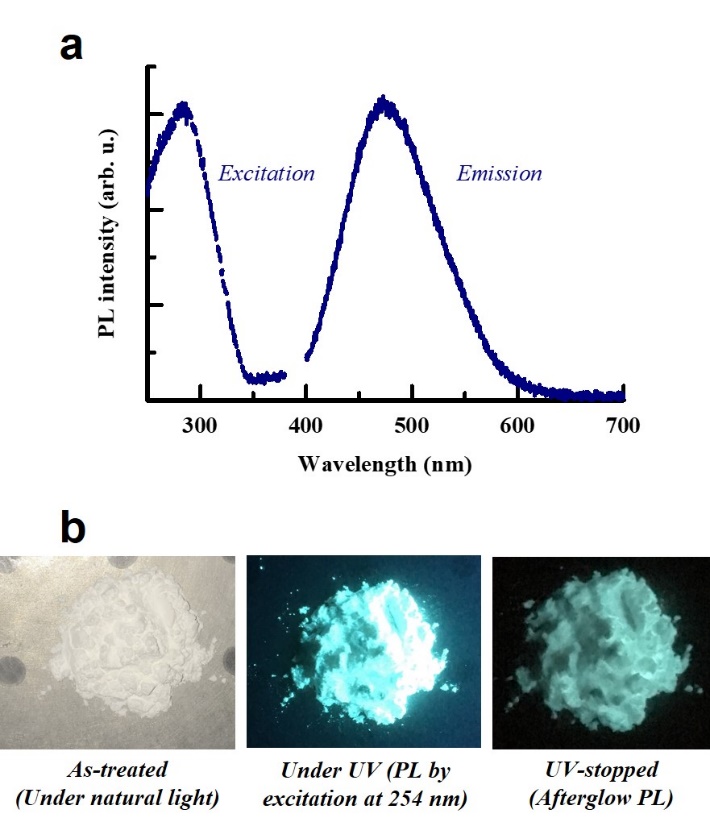


**Fig. S2.** (a) PL and PLE spectra in the ZrO_2_ sample prepared in this study. Commercial ZrO_2_ powder is subjected to isothermal treatment at 1400ºC for 6 h (see text). (b) Pictures of the as-treated ZrO_2_ sample, the sample under UV-irradiation, and the sample after the UV-stopped. Clear visible-PL and afterglow could be seen.


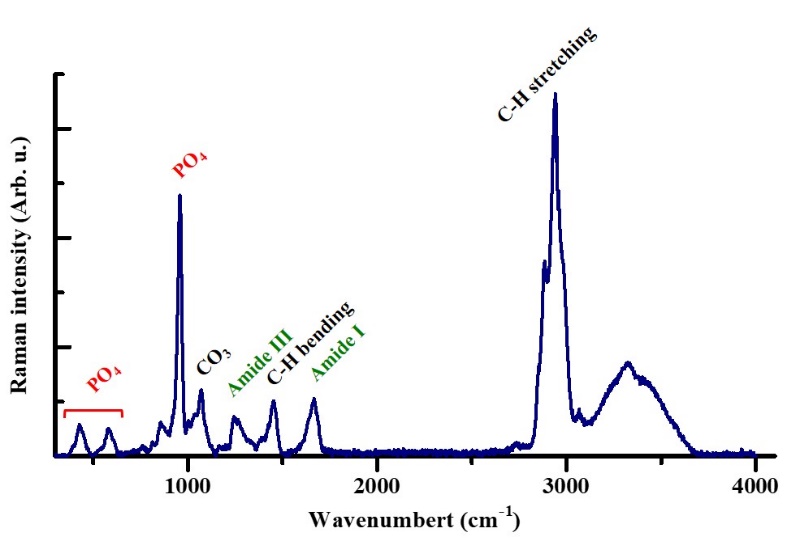


**Fig. S3.** Raman spectrum of the bone sample in this study. The major Raman bands can be assigned by comparison to the spectral data of human bone so far [21,22] because of the significant spectral analogies: The Raman bands at ~430 cm^−1^, ~580 cm^−1^, and ~960 cm^−1^ are ascribed to phosphate vibrations. The bands at ~1070 cm^−1^ is due to the carbonate substitution for phosphate in the apatite lattice. The bands at ~1250 cm^−1^ and 1670 cm^−1^ are ascribed to amide III and amide I, respectively. The bands at ~1450 cm^−1^ and 2940 cm^−1^ is related to the bending and stretching modes of C−H bonds, respectively.
